# Palmitoylethanolamide: A Multifunctional Molecule for Neuroprotection, Chronic Pain, and Immune Modulation

**DOI:** 10.3390/biomedicines13061271

**Published:** 2025-05-22

**Authors:** Valeria Di Stefano, Luca Steardo, Martina D’Angelo, Francesco Monaco, Luca Steardo

**Affiliations:** 1 Department of Health Sciences, University of Catanzaro Magna Graecia, 88100 Catanzaro, Italy; valeria.distefano@studenti.unicz.it (V.D.S.); martina.dangelo001@studenti.unicz.it (M.D.); 2 Department of Mental Health, Azienda Sanitaria Locale Salerno, 84132 Salerno, Italy; fmonaco1980@gmail.com; 3 European Biomedical Research Institute of Salerno (EBRIS), 84125 Salerno, Italy; 4 Department of Physiology and Pharmacology “Vittorio Erspamer”, Sapienza University of Rome, 00185 Rome, Italy; luca.steardo@uniroma1.it; 5 Department of Clinical Psychology, University Giustino Fortunato, 82100 Benevento, Italy

**Keywords:** palmitoylethanolamide, neuroprotection, chronic pain, endocannabinoid system, immune modulation

## Abstract

Palmitoylethanolamide (PEA) is an endogenous lipid mediator belonging to the N-acyl-ethanolamine family, widely recognized for its multifaceted effects on neuroprotection, chronic pain management, and immune modulation. As a naturally occurring compound, PEA plays a crucial role in maintaining homeostasis under conditions of cellular stress and inflammation. Its pharmacological effects are primarily mediated through peroxisome proliferator-activated receptor-alpha (PPAR-α) activation, alongside indirect modulation of cannabinoid receptors CB1 and CB2, as well as interactions with novel targets such as GPR55 and TRPV1. These molecular mechanisms underpin its broad therapeutic potential, particularly in the management of neuroinflammatory and neurodegenerative disorders, pain syndromes, and immune dysregulation. A major advancement in PEA research has been the development of ultramicronized palmitoylethanolamide (umPEA), which significantly enhances its bioavailability and therapeutic efficacy by facilitating better tissue absorption and interaction with key molecular pathways. Preclinical and clinical studies have demonstrated that umPEA is particularly effective in reducing neuroinflammation, stabilizing mast cells, and enhancing endocannabinoid system activity, making it a promising candidate for integrative approaches in neuropsychiatric and chronic inflammatory diseases. Given its well-established safety profile, umPEA represents an attractive alternative or adjunct to conventional anti-inflammatory and analgesic therapies. This communication provides a comprehensive overview of the mechanisms of action and therapeutic applications of both PEA and umPEA, emphasizing their emerging role in clinical practice and personalized medicine.

## 1. Introduction

The increasing focus on integrative approaches for the management of neuroinflammatory and immune-related disorders has prompted the investigation of natural compounds with potential modulatory effects. Among these, palmitoylethanolamide (PEA), especially in its ultramicronized palmitoylethanolamide (umPEA), is gaining recognition as a promising therapeutic option for these conditions. Based on growing preclinical and clinical evidence, we argue that umPEA deserves greater consideration within therapeutic protocols, especially in neuropsychiatric care and personalized medicine. PEA, an endogenous bioactive lipid mediator belonging to the N-acyl-ethanolamine (NAE) family, has garnered significant attention due to its diverse pharmacological properties. In response to cellular stress or injury, this compound is synthesized on demand within the lipid bilayer and exerts its effects locally within tissues, including the brain. PEA is upregulated in pathological conditions, indicating its role as a protective and homeostatic agent. Its broad spectrum of biological activities includes anti-inflammatory, analgesic, anticonvulsant, antimicrobial, antipyretic, antiepileptic, immunoregulatory, and neuroprotective effects [1].

PEA is synthesized on demand within the lipid bilayer via a two-step enzymatic process. The initial step involves a calcium- and cAMP-dependent transfer of palmitic acid from phosphatidylcholine to phosphatidylethanolamine, generating N-acylphosphatidylethanolamine (NAPE), which is then hydrolyzed by an NAPE-specific phospholipase D to release PEA. Its degradation is mediated by fatty acid amide hydrolase (FAAH) and a PEA-preferring acid amidase (PAA), both converting PEA into palmitic acid and ethanolamine. Beyond its direct effects, PEA also modulates the endocannabinoid system by inhibiting FAAH, the enzyme responsible for degrading anandamide (AEA). As a result, AEA levels increase, promoting greater activation of cannabinoid receptors CB1 and CB2 and contributing to anti-inflammatory and analgesic actions through the so-called “entourage effect” [2]. PEA has been detected in virtually all mammalian tissues, including the brain. Although the regulation of its endogenous levels is not yet fully understood, several studies indicate that PEA concentrations rise in response to tissue injury and cellular stress. This supports the notion that PEA functions as an intrinsic protective agent, mobilized to restore local homeostasis and counteract inflammation [3]. For instance, in response to cellular damage or tissue injury, macrophages, mast cells, and keratinocytes are known to release PEA as part of the body’s natural response to mitigate inflammation and promote healing [4]. In the brain, PEA has been shown to increase in conditions including neurodegenerative diseases such as Alzheimer’s disease and Parkinson’s disease, as well as multiple sclerosis and traumatic brain injury [5,6]. PEA also exerts neuroprotective effects, protecting neuronal cells from oxidative damage and neurodegeneration [7,8].

The therapeutic potential of PEA is driven by its interactions with several molecular targets. A primary target is PPAR-α, a nuclear receptor involved in regulating lipid metabolism and inflammation [9]. By promoting fatty acid oxidation and reducing pro-inflammatory cytokines like TNF-α, IL-1β, and IL-6, PEA helps manage conditions such as metabolic syndrome and cardiovascular diseases, highlighting its role in correcting lipid dysregulation and chronic inflammation [10].

The increasing prevalence of chronic degenerative diseases is a major global health concern, driven in part by the nutrition transition, a shift in dietary and lifestyle patterns toward the consumption of highly processed foods, sedentary behaviors, and exposure to environmental stressors. These changes have led to widespread metabolic imbalances, chronic inflammation, and an overall decline in health, predisposing individuals to a range of chronic conditions such as metabolic syndrome, cardiovascular diseases, neurodegenerative disorders, gastrointestinal diseases, and autoimmune conditions [11,12,13,14].

Despite advancements in pharmacological treatments, the toxicity and side effects associated with long-term drug use limit their application in preventive medicine [15]. Therefore, an urgent need is to explore alternative strategies to restore metabolic homeostasis and improve public health outcomes [16]. Nutritional supplementation has emerged as a promising avenue for recreating pre-industrial dietary profiles and mitigating the impact of modern dietary insufficiencies [17,18,19,20]. Ideal supplements should possess anti-inflammatory, antioxidant, immunomodulatory, neuroprotective, and analgesic properties while enhancing cognitive function and overall recovery processes [21].

Additionally, PEA activates TRPV1, a receptor involved in pain perception and neuroinflammation. This activation leads to desensitization of sensory neurons, resulting in reduced pain. Additionally, PEA stabilizes mast cells, reducing allergic and inflammatory responses, underscoring its potential in treating neuropathic pain and chronic pain syndromes [22,23]. PEA also interacts with GPR55 and GPR119, two GPCRs involved in lipid metabolism and pain modulation. While the exact mechanisms are still under investigation, these interactions suggest additional pathways through which PEA may exert therapeutic effects, particularly in metabolic and neurodegenerative diseases [24].

The therapeutic versatility of PEA, particularly in its umPEA, has been demonstrated across a broad spectrum of conditions, including allergic and respiratory disorders, viral infections, chronic pain syndromes, musculoskeletal diseases, psychiatric illnesses, and neurodegenerative disorders [25]. Moreover, umPEA supplementation has been associated with enhanced muscle recovery, improved cognitive performance, mood stabilization, and better sleep regulation [26]. Exogenous administration offers a promising strategy for restoring physiological balance since endogenous PEA levels are often insufficient to counteract the persistent inflammatory stress linked to chronic diseases [4].

Currently, several PEA-containing formulations are available as nutraceuticals, dietary supplements, or medical foods in various countries; however, the ultra-micronized formulation of PEA alone can cross biological barriers, including the blood–brain barrier, thereby ensuring therapeutically active concentrations [27]. These products are typically administered at a recommended daily dosage of 1200 mg [28]. Notably, PEA’s long-established safety profile, supported by both clinical and preclinical research dating back to the 1950s, further reinforces its potential as a valuable adjunctive therapy for managing chronic health conditions [29]. Considering the growing scientific evidence on the role of PEA in modulating inflammation and pain, this communication aims to summarize its key mechanisms of action and explore its potential clinical applications. Specifically, we will discuss the value of PEA, and particularly umPEA, as a safe and effective therapeutic option for chronic inflammatory conditions, neurodegenerative diseases, and immune system disorders.

## 2. Ultramicronized Palmitoylethanolamide

umPEA is a specifically engineered formulation of PEA, obtained by subjecting standard PEA to a controlled ultramicronization process. Ultramicronization involves mechanically reducing the particle size of PEA crystals to dimensions typically below 10 μm. This significant reduction in particle size increases the surface area of the compound, thereby markedly improving dissolution rates and facilitating rapid and enhanced absorption through the gastrointestinal mucosa [30]. These pharmacological enhancements directly translate into greater clinical effectiveness, allowing lower effective doses and yielding improved therapeutic outcomes across various inflammatory, neuropathic, and neurodegenerative conditions [31,32,33]. Such advantages have been validated by multiple pharmacokinetic studies and clinical trials, reinforcing the therapeutic superiority of umPEA in clinical practice.

### Pharmacokinetic and Pharmacodynamic Advantages of umPEA

Standard formulations of PEA are characterized by limited oral bioavailability and suboptimal tissue distribution, restricting their therapeutic effectiveness. umPEA, obtained through an advanced ultramicronization process that reduces particle size to the micrometer scale, significantly enhances PEA’s pharmacokinetic profile [3]. The ultramicronization markedly increases gastrointestinal absorption, leading to higher systemic bioavailability and elevated tissue concentrations. Crucially, this formulation allows umPEA to more effectively cross biological barriers, including the blood–brain barrier, thus achieving therapeutically relevant concentrations within the central nervous system [34]. Consequently, umPEA demonstrates superior pharmacodynamic activity compared to non-micronized PEA, providing enhanced efficacy in reducing neuroinflammation, stabilizing mast cells, modulating glial cell activity, and potentiating endogenous endocannabinoid system function [4]. These pharmacokinetic and pharmacodynamic improvements position umPEA as a clinically preferable alternative, especially for chronic inflammatory, neuropathic, and neuropsychiatric conditions, supported by robust preclinical and emerging clinical evidence [35].

## 3. PEA and Immunity

### 3.1. PEA and Immunity

PEA has long been recognized for its immunomodulatory properties, initially highlighted in clinical studies from the 1970s demonstrating its efficacy in reducing the incidence and severity of viral respiratory infections [36]. umPEA enhances nonspecific immune resistance against pathogens while balancing immune responses and mitigating excessive inflammation. Its immunomodulatory action primarily involves modulation of innate immune cells such as mast cells, macrophages, and microglia, preventing excessive activation and consequent neuroinflammation. By reducing cytokine release, stabilizing mast cells, and promoting microglial homeostasis, umPEA acts as an effective neuroprotective and immunomodulatory agent without the adverse effects associated with classical immunosuppressants [37,38]. Preclinical studies underscore its efficacy in conditions characterized by neuroinflammation, including Alzheimer’s, Parkinson’s, and traumatic brain injury, highlighting its potential as a therapeutic strategy for immune-mediated neurodegenerative disorders [37,38,39]. PEA’s extensive immunomodulatory effects, supported by numerous studies, highlight its ability to influence multiple physiological pathways in a coordinated manner to achieve therapeutic benefits. Due to its well-documented safety profile, PEA holds promise as a prophylactic agent for maintaining immune health and supporting overall well-being [40]. Unlike other endocannabinoids, its metabolic breakdown produces non-toxic byproducts, making it suitable for long-term use without significant adverse effects. Incorporating umPEA into health and wellness programs may serve as a viable approach to promoting immune resilience and fostering healthy aging [41].

### 3.2. Modulation of the Gut–Brain Axis and Gut-Derived Immunity by PEA

PEA contributes significantly to gut homeostasis and gut-derived immune regulation, primarily through its interaction with PPAR-α receptors, enhancing intestinal barrier integrity and mitigating local inflammation [42]. By activating CB2 receptors, PEA reduces gut-derived pro-inflammatory cytokines, limiting systemic inflammation and associated neurological consequences [43]. In experimental colitis models, umPEA reduced intestinal inflammation and immune cell infiltration and promoted epithelial regeneration [42,44]. Additionally, PEA may influence gut–brain interactions via GPR119 receptors, stimulating the secretion of glucagon-like peptide-1 (GLP-1), thus contributing to cognitive and emotional stability [1]. These mechanisms highlight umPEA’s potential therapeutic value in disorders involving gut dysbiosis, inflammation, and neuroimmune dysfunction [45,46,47,48].

## 4. PEA and Inflammatory Reactions

### 4.1. PEA’s Antiallergic Effects

Allergic conditions, including allergic rhinitis, dermatitis, and asthma, are primarily characterized by excessive inflammatory responses and the infiltration of immune cells into affected tissues [49]. Upon exposure to allergens, mast cells become activated and release various inflammatory mediators, such as histamine, cytokines, and chemokines, leading to symptom manifestation [50].

The antiallergic potential of PEA was first noted in the mid-20th century when early studies suggested its ability to mitigate allergic reactions. Subsequent research has confirmed PEA’s capacity to modulate immune cell responses, particularly by reducing mast cell activation and degranulation [36]. These protective effects are largely attributed to its ability to interact with specific cellular targets, including PPAR-α and GPR55 receptors, as well as its indirect influence on CB1, CB2, and TRPV1 receptors.

Experimental models have provided compelling evidence of PEA’s efficacy in reducing allergic inflammation [37]. Studies on canine skin mast cells demonstrated that PEA administration significantly inhibited the release of pro-inflammatory mediators such as prostaglandin D2, tumor necrosis factor-alpha (TNF-α), and histamine in a dose-dependent manner [40]. Similarly, in hypersensitive canine models, oral administration of umPEA effectively reduced allergic skin reactions, while in feline models, it alleviated symptoms associated with eosinophilic granuloma. Other research has shown that PEA supplementation helps manage atopic dermatitis and pruritus in dogs by reducing mast cell recruitment and inflammatory responses [38].

In murine models of allergic airway inflammation, reduced bronchial PEA levels have been observed alongside increased CB2 and GPR55 receptor expression [24]. Supplementation with PEA before allergen exposure significantly inhibited leukocyte infiltration, pulmonary inflammation, mast cell recruitment, and leukotriene production, suggesting a regulatory role in airway hypersensitivity reactions. Additionally, experimental models of contact allergic dermatitis have shown that umPEA exerts stronger anti-inflammatory effects compared to standard PEA. In these models, umPEA reduced mast cell numbers and limited angiogenic responses associated with hypersensitivity, indicating its enhanced therapeutic potential [39].

The key difference between PEA and umPEA lies in their bioavailability and efficacy. While both forms of PEA share similar mechanisms of action—modulating mast cell activity, inflammatory mediator release, and immune responses—the ultramicronization process of umPEA significantly improves its absorption and bioavailability. While both forms of PEA share similar mechanisms of action—modulating mast cell activity, inflammatory mediator release, and immune responses—the ultramicronization of PEA markedly enhances its absorption and bioavailability, leading to higher tissue concentrations and improved therapeutic outcomes. This enhanced bioavailability enables umPEA to provide superior efficacy in alleviating symptoms associated with allergic conditions. Preclinical and clinical evidence suggests that umPEA, due to its optimized pharmacokinetic profile, offers a more potent therapeutic solution for the management of chronic inflammatory diseases, including allergic dermatitis and asthma, compared to standard PEA formulations [3,51]. Limited clinical studies in humans have also indicated the potential benefits of PEA in allergic conditions. While these findings are promising, further large-scale clinical trials are needed to fully establish the efficacy of PEA in managing allergic and inflammatory disorders [46,47].

### 4.2. PEA and Brain Health

Neuroinflammation is increasingly recognized as a fundamental contributor to the development and progression of numerous neurodegenerative diseases, including Alzheimer’s disease, Parkinson’s disease, stroke, traumatic brain injury (TBI), autism spectrum disorder (ASD), epilepsy, and various cognitive, behavioral, and mood disorders. The presence of chronic inflammation in the central nervous system (CNS) can lead to the progressive deterioration of neuronal structures, impairing function and ultimately contributing to disease pathology [5,8,42].

Key players in neuroinflammatory processes include mast cells, microglia, and astrocytes, which, when persistently activated, release inflammatory mediators that exacerbate neuronal damage [43]. Although the detailed molecular mechanisms of neuroinflammation are complex, it is widely accepted that chronic activation of these pathways disrupts neuronal homeostasis and accelerates neurodegenerative processes. Consequently, therapeutic strategies aimed at mitigating neuroinflammation hold promise for preserving brain health and function [48].

Exogenous administration of umPEA has been explored in preclinical and clinical settings, where it has demonstrated beneficial effects in conditions such as stroke, neurotrauma, and neurodegenerative diseases. By enhancing endogenous neuroprotective mechanisms and dampening harmful inflammatory cascades, umPEA may support long-term brain health and function [25,44].

Moreover, recent meta-analytical evidence further supports the cognitive benefits of PEA supplementation. Specifically, Colizzi et al. (2022), in their systematic review and preliminary meta-analysis, demonstrated that PEA significantly improves cognitive function, as evidenced by enhanced Mini-Mental State Examination (MMSE) scores of 3.80 points (95% CI: −0.16 to 7.75), indicating a trend toward cognitive enhancement, and positively affects executive function, working memory, language deficits, and daily living activities. These findings strongly reinforce the therapeutic potential of PEA in neurocognitive disorders, suggesting its role not merely in symptom management but potentially in slowing cognitive decline progression [52].

Additionally, PEA has shown efficacy in mitigating negative symptoms commonly associated with psychotic and manic disorders, including schizophrenia and bipolar disorder, supporting its tolerability and possible use as a safe and effective therapeutic intervention in the psychiatric setting [53].

Given the growing body of research supporting umPEA’s role in neuroinflammation and neuroprotection, its application in the management of neurodegenerative diseases and neurological disorders is an area of increasing interest [54,55]. Further studies are needed to fully elucidate its mechanisms of action, optimal dosing strategies, and long-term efficacy in maintaining cognitive function and preventing neurodegeneration.

### 4.3. PEA’s Effects on Mood, Cognition, and Behavior

PEA’s role extends beyond inflammation but also has a direct impact on mood, anxiety, stress, and cognitive function through its interactions with the endocannabinoid system and other neuromodulatory pathways. The significance of endocannabinoids and N-acyl-ethanolamines in regulating cognitive function, mood, and behavior is well-established [56,57]. Endogenous PEA levels have been shown to increase in response to acute psychosocial stress, acting as an adaptive mechanism to counteract inflammatory and excitotoxic cascades within the central nervous system, as happens in PTSD and depression. Interestingly, individuals with PTSD often self-medicate with cannabis, possibly due to its effects on the endocannabinoid system [58]. Higher PEA concentrations have been observed in affected individuals, correlating with symptom severity [59]. This suggests that while PEA is upregulated as part of an adaptive response, endogenous levels may be insufficient or depleted to counteract chronic stress, proposing that supplementation could help restore neurochemical balance [60,61].

At the same time, antidepressants have been found to increase brain PEA levels, further supporting its relevance in mood regulation [62]. Furthermore, in patients with major depressive disorder, the addition of umPEA to standard antidepressant treatment led to significantly greater improvements in depressive symptoms compared to placebo. In animal models of Alzheimer’s disease and traumatic brain injury, PEA supplementation has been shown to improve memory function while reducing anxiety, aggression, and depressive symptoms [25,63]. Reports from case studies on individuals across the autism spectrum, both children and adults, suggest that PEA supplementation improves social behaviors, cognitive function, and overall adaptability [64,65].

Instead, umPEA is more effective on the CNS due to its ability to cross the blood–brain barrier.

The therapeutic potential of umPEA in these neuropsychiatric conditions is increasingly being explored in preclinical and clinical studies that have shown promising beneficial effects (personal data, paper in preparation).

Studies have demonstrated that umPEA, in its micronized form, exhibits enhanced pharmacokinetic properties, including improved bioavailability and more efficient distribution within the CNS compared to conventional PEA [66]. This enhanced profile makes umPEA particularly advantageous for conditions characterized by neuroinflammation, neurodegeneration, or chronic nociceptive and neuropathic pain. The ability of umPEA to effectively cross the blood–brain barrier and achieve central distribution allows it to directly interact with the brain and spinal cord, enhancing its therapeutic potential for disorders such as neuroinflammatory and neurodegenerative conditions [67].

PEA’s ability to support neurogenesis and synaptic remodeling likely plays a fundamental role in its effects on cognition and behavior. Moreover, as chronic pain and mood disorders negatively impact memory and executive function, PEA’s concurrent antidepressant, anxiolytic, and analgesic properties may contribute to its cognitive-enhancing potential. Given its ability to target neuroinflammation, modulate pain perception, alleviate depressive symptoms, and promote neuroplasticity, umPEA emerges as a promising therapeutic candidate for neuropsychiatric and neurocognitive disorders [68]. While current findings are encouraging, further clinical trials are necessary to establish optimal dosing strategies and confirm their long-term benefits in both clinical populations and healthy individuals aiming to support brain health.

### 4.4. Inflammation and Pain

Inflammation plays a crucial role in pain, serving both as a protective and a pathological mechanism. While acute inflammation is essential for tissue healing and defense against injury, chronic inflammation contributes to the persistence of pain and the development of neuropathic conditions [69]. Non-neuronal cells, such as mast cells, microglia, and astrocytes, play a key role in amplifying and sustaining inflammatory responses, sensitizing pain pathways. This interaction leads to central and peripheral sensitization, where normally non-painful stimuli are perceived as painful [70].

#### 4.4.1. umPEA Mechanism of Action

Inflammatory pain refers to pain resulting from tissue injury and the associated immune response, typically characterized by the activation of peripheral nociceptors due to the release of pro-inflammatory mediators such as cytokines and prostaglandins. Persistent pain, often overlapping with chronic pain, is defined as pain that lasts beyond normal tissue healing time, generally more than three months, and may involve continued inflammation, sensitization of neural pathways, or maladaptive neuroimmune mechanisms [71].

Palmitoylethanolamide (umPEA), an endogenous lipid molecule, plays a key role in pain relief by modulating this neuroimmune response. It activates PPAR-α and GPR55 receptors and exerts indirect effects on CB1, CB2, and TRPV1 receptors. By inhibiting mast cell activation, umPEA suppresses the release of key pro-inflammatory mediators such as nerve growth factor (NGF), cyclooxygenase-2 (COX-2), tumor necrosis factor-alpha (TNF-α), and inducible nitric oxide synthase (iNOS) [10]. Additionally, it reduces microglial and astrocyte activation, preserving peripheral nerve morphology, reducing endoneurial edema, and limiting macrophage infiltration in inflamed tissues.

#### 4.4.2. Differences in umPEA Action Across Pain Types

Pain syndromes can be broadly classified into nociceptive, neuropathic, and nociplastic pain, each characterized by distinct pathophysiological mechanisms. Nociceptive pain arises from tissue injury and inflammation, triggering peripheral nociceptors. umPEA addresses this pain type primarily through its anti-inflammatory properties, stabilizing mast cells, and inhibiting cytokine release. Neuropathic pain, resulting from damage or dysfunction within the nervous system itself, involves neuroinflammation and sensitization of neural pathways [72]. umPEA exerts its therapeutic effects in neuropathic conditions by modulating microglial activation, reducing pro-inflammatory cytokines, and normalizing sensory neuron excitability via interactions with PPAR-α, CB2, and TRPV1 receptors [10,73]. Finally, nociplastic pain represents pain without clear tissue or nerve injury, largely driven by abnormal central processing of pain signals. Here, umPEA’s ability to cross the blood–brain barrier, modulate central glial cells, and enhance endogenous analgesic mechanisms through the endocannabinoid system is particularly beneficial [23,32,74]. Thus, umPEA offers targeted, multimodal relief tailored to the specific underlying mechanisms of each distinct pain type.

#### 4.4.3. Preclinical and Clinical Evidence

Preclinical and clinical studies have documented the efficacy of umPEA in the treatment of inflammatory and chronic pain, with applications in conditions such as fibromyalgia, osteoarthritis, postoperative pain, and endometriosis [75,76,77]. In cases of chronic pain, endogenous PEA levels may be reduced, but supplementing with exogenous umPEA can restore its protective and anti-inflammatory effects [70].

#### 4.4.4. Comparison with Pharmaceutical Analgesics

Although conventional analgesic medications, such as NSAIDs, opioids, and corticosteroids, are commonly used for chronic pain, their long-term use carries significant risks of gastrointestinal, hepatic, cardiovascular, and renal complications [78]. In contrast, umPEA stands out for its favorable safety profile, offering a promising, natural, and well-tolerated alternative for individuals suffering from chronic pain and inflammation. Further clinical research is necessary to optimize therapeutic strategies and dosing for long-term pain management [79]. A comparative overview of the pharmacological and clinical advantages of standard PEA versus umPEA is summarized in Table 1.

### 4.5. PEA’s Effects on Primary Headaches

Headaches are one of the most prevalent neurological disorders, affecting nearly all individuals at some point in their lives. Among these, primary headaches, including migraines and tension-type headaches (TTH), constitute a significant global health burden. These conditions can occur as isolated episodic events or develop into chronic disorders, severely impacting quality of life and daily functioning [82,83].

Chronic TTH is believed to involve central sensitization and a deficiency in endogenous pain control mechanisms, with nociceptive input originating from myofascial tissues around the cranium. In migraines, early stages are thought to be triggered by the activation of trigeminal nociceptors due to neurovascular inflammation in the meninges and cranial blood vessels [84]. Once sensitization occurs in the perivascular trigeminal nerve terminals, non-painful stimuli can provoke pain responses. Moreover, dural mast cells and associated glial cells contribute to migraine pathology by releasing pronociceptive and pro-inflammatory mediators, which further drive sensitization [85,86].

umPEA has emerged as a potential therapeutic agent for primary headaches due to its ability to modulate inflammation and pain signaling. By regulating the release of inflammatory mediators and stabilizing mast cells, umPEA may help reduce the excessive nociceptive input that contributes to headache pathophysiology [87]. Clinical studies have explored the efficacy of umPEA in treating migraines [88,89]. Research has demonstrated that sublingual administration of PEA leads to a reduction in headache frequency, duration, and intensity, as well as a decrease in the use of analgesic medications among individuals with migraine [88]. Additionally, PEA supplementation has shown benefits in patients with migraines with aura who were concurrently using non-steroidal anti-inflammatory drugs (NSAIDs), leading to significant reductions in pain severity, attack frequency, and duration. Similar findings have been observed in pediatric migraine populations, where PEA supplementation resulted in a marked decrease in attack frequency and severity over a three-month period [89].

These findings suggest that PEA holds promise not only as a migraine prophylactic but also as a potential therapeutic option for tension-type headaches. Its ability to regulate neuroinflammation, stabilize nociceptive signaling pathways, and support endogenous pain control mechanisms makes it an attractive candidate for future headache management strategies [9]. Further clinical trials are necessary to establish optimal dosing and long-term efficacy in broader populations.

### 4.6. PEA and Sleep

Chronic pain is particularly known to interfere with sleep quality, with conditions such as neuropathic pain, osteoarthritis, diabetic neuropathy, and carpal tunnel syndrome being strongly associated with sleep disruptions. Circadian rhythms regulate numerous essential physiological processes, including sleep–wake cycles, cognitive performance, and overall homeostasis [90]. Disruptions to these rhythms can negatively impact both physical and mental well-being, leading to increased sympathetic nervous system activity, stress susceptibility, mood disorders, and cognitive decline [91]. Persistent disturbances in sleep patterns have been linked to impairments in synaptic plasticity, deficits in attention and working memory, and increased susceptibility to depression and neurodegenerative conditions [92].

Various external and internal factors, including environmental stressors, psychosocial pressures, and underlying medical conditions, can contribute to sleep disturbances [93]. While prescription sedatives and tranquilizers are commonly used to manage sleep disorders, they come with risks of dependence and adverse side effects [94,95]. Cannabinoid-based treatments offer an alternative, but prolonged use may result in withdrawal-induced sleep deprivation, underscoring the need for natural, well-tolerated sleep aids [96,97].

#### PEA’s Role in Sleep Regulation

Given the close interplay between pain, stress, and sleep disturbances, umPEA’s analgesic and anxiolytic properties make it a promising candidate for improving sleep quality [69]. By modulating TRPV1 channels and enhancing anandamide signaling via the entourage effect, PEA may help alleviate sleep disturbances associated with pain and anxiety. Additionally, its ability to modulate the hypothalamic–pituitary–adrenal (HPA) axis may mitigate stress-induced sleep disorders.

Emerging clinical research supports umPEA’s role in improving sleep patterns. In individuals experiencing chronic pain, PEA supplementation has been shown to reduce stress, anxiety, and associated sleep disruptions [70]. A study involving patients with osteoarthritis demonstrated that PEA not only alleviated joint pain but also improved overall sleep quality [98]. Furthermore, in patients recovering from carpal tunnel syndrome surgery, PEA supplementation significantly enhanced sleep–wake rhythms and overall restfulness [99].

Declining levels of anandamide have been associated with sleep disturbances in aging populations [100]. Through its role in supporting endocannabinoid signaling, umPEA may help maintain circadian balance and cognitive function in both healthy individuals and those at risk for sleep disorders. While further research is needed to fully elucidate its long-term benefits, PEA presents a compelling option for promoting restorative sleep and overall well-being without the adverse effects associated with conventional sleep aids.

## 5. Limitations

Despite growing interest in PEA, especially umPEA, several limitations persist. The current evidence is primarily based on heterogeneous preclinical studies and small-scale clinical trials, often lacking robust designs such as adequate randomization and control groups, which compromises validity and generalizability [25]. Additionally, significant variability in formulations, dosing regimens, and study outcomes hampers the comparability and reproducibility of results, limiting definitive conclusions and robust meta-analyses [29]. To establish umPEA’s therapeutic role clearly, future studies must include larger, randomized controlled trials and standardized protocols addressing pharmacokinetic variability and clinical efficacy across diverse populations [99].

## 6. Conclusions and Future Perspectives: The Anti-Inflammatory Potential of PEA in Clinical Practice

PEA, and particularly its umPEA, has demonstrated significant potential as a safe and effective anti-inflammatory agent with broad applications in the management of neurodegenerative diseases, chronic pain syndromes, and immune-related disorders [101]. Its multimodal mechanism of action via PPAR-α activation, mast cell stabilization, and indirect modulation of the endocannabinoid system confers a unique therapeutic versatility that distinguishes it from conventional anti-inflammatory and analgesic drugs [102].

Unlike NSAIDs or corticosteroids, umPEA does not carry the risk of gastrointestinal bleeding, cardiovascular complications, or renal impairment, which often limit the long-term use of traditional agents in chronic conditions [103]. Moreover, its favorable tolerability profile, supported by decades of clinical and preclinical data, renders it a compelling candidate for long-term, integrative treatment strategies in both preventive and therapeutic contexts.

In our opinion, umPEA is not merely a complementary option—it represents a paradigm shift in how we approach chronic inflammation, particularly in neuropsychiatric and pain-related disorders. We believe its incorporation into clinical protocols should be actively pursued, especially in patients with poor tolerability to standard treatments or in those with complex, multisystemic symptoms.

Nonetheless, the integration of umPEA into mainstream medical practice requires a concerted effort to overcome regulatory inertia, establish standardized guidelines, and generate high-quality evidence through rigorous clinical trials. In this regard, we strongly advocate for a translational research agenda that prioritizes head-to-head comparisons, personalized dosing strategies, and mechanistic studies aimed at identifying predictive biomarkers of response.

In summary, we believe that umPEA, by virtue of its safety, pleiotropic effects, and neuroimmune targeting capacity, should be considered not only as an adjunct but as a central pillar in the future of anti-inflammatory therapy, particularly within the framework of precision medicine.

Given the accumulating preclinical and clinical evidence, and in light of its excellent safety profile, we urge clinicians and researchers to reconsider the role of umPEA not as a marginal nutraceutical but as a scientifically grounded therapeutic agent deserving full clinical dignity. We advocate for its inclusion in clinical guidelines, particularly for complex syndromes where inflammation, neuroglial dysfunction, and immune dysregulation converge. In an era that increasingly demands personalized, multimodal, and well-tolerated therapies, umPEA represents an underutilized asset. The time has come to move beyond skepticism and integrate this molecule into the therapeutic arsenal with the scientific rigor and clinical openness it deserves.

## 7. Practical Clinical Recommendations for umPEA Use

As a well-tolerated, pleiotropic molecule acting across neuroimmune, glial, and metabolic pathways, **umPEA** should be actively considered in the clinical management of complex chronic conditions. Its use is particularly valuable when conventional therapies are limited by adverse effects, polypharmacy, or suboptimal response.

### 7.1. Chronic Pain and Inflammatory Disorders

- **Neuropathic Pain and Fibromyalgia**. umPEA should be used as an adjunct to gabapentinoids or antidepressants to enhance analgesia while minimizing dose-dependent side effects. A starting dose of **600 mg twice daily** is recommended, with individual titration based on clinical response [77,80,104,105].
- **Autoimmune and Systemic Inflammation**. In diseases such as **rheumatoid arthritis** or **inflammatory bowel disease**, umPEA should be considered as a supportive agent to downregulate immune hyperactivity and systemic inflammation, particularly in patients with contraindications to long-term steroid or NSAID use [77,106].

### 7.2. Neuroinflammation and Neurodegenerative Conditions

- **Alzheimer’s and Parkinson’s Disease**. Given its neuroprotective and anti-inflammatory actions, umPEA (**600–1200 mg/day**) is a rational adjunct in early-phase disease management aimed at slowing cognitive and functional decline [5,25].
- **Stroke and Traumatic Brain Injury (TBI)**. Post-acute supplementation with umPEA (**600 mg twice daily**) may enhance recovery, reduce neuroinflammation, and promote neuroplasticity, as supported by preclinical and emerging clinical evidence [107,108].

### 7.3. Gut–Brain Axis and Immune Regulation

- **Leaky Gut and Microbiota Dysregulation**. umPEA (**300–600 mg/day**) in combination with targeted probiotics can restore gut barrier function, mitigate dysbiosis, and reduce systemic endotoxemia, especially in IBS, Crohn’s disease, or colitis [73,109].
- **Immune Support and Viral Infections**. Historical and experimental data support the use of umPEA for enhancing non-specific immune defense. **600 mg/day** is recommended for immune support, with **1200 mg/day** during acute respiratory infections or high-risk exposure periods [110].

The clinical indications are summarized in Table 2.

Table 3 details the observed clinical outcomes associated with umPEA administration in different conditions, highlighting model types and key mechanisms involved.

## 8. Final Considerations

In our view, umPEA should not be relegated to the margins of integrative medicine but rather promoted as a core tool in personalized anti-inflammatory strategies. Its exceptional tolerability, combined with a multimodal mechanism of action, makes it uniquely suited for long-term management of inflammation-driven disorders. While additional large-scale RCTs are warranted to refine dosage and indications, the current evidence justifies its immediate, judicious incorporation into clinical algorithms, particularly in patients for whom conventional therapies have proven insufficient or intolerable. Although pharmacokinetic and preclinical studies have demonstrated superior bioavailability and tissue distribution of micronized and ultra-micronized PEA compared to naïve formulations, direct head-to-head clinical trials comparing umPEA with other PEA formulations remain limited, highlighting the need for further randomized controlled studies [22,112,113]

**Table 1 Legend:** Comparison of pharmacological and clinical differences between standard PEA and ultramicronized PEA (umPEA). The ultramicronization process significantly reduces particle size (<10 µm), greatly enhancing oral bioavailability, systemic absorption, and blood–brain barrier penetration. Consequently, umPEA achieves higher tissue concentrations, faster therapeutic onset, and greater clinical efficacy at lower doses compared to standard PEA formulations. These pharmacokinetic and pharmacodynamic advantages position umPEA as a preferable therapeutic option for inflammatory, neuropathic, and neurodegenerative conditions.

## Figures and Tables

**Table 1:** Comparison of standard PEA and umPEA: key pharmacological and clinical differences.

Aspect	Standard PEA	Ultramicronized PEA (umPEA)
**Particle Size**	Large particles (>100 µm)	Reduced particles (<10 µm)
**Bioavailability**	Low oral bioavailability, slow absorption	Enhanced oral bioavailability, rapid absorption
**Blood–brain barrier penetration**	Limited CNS penetration	Improved CNS penetration
**Pharmacokinetics**	Lower peak plasma and tissue concentrations	Higher peak plasma and tissue concentrations
**Onset of Action**	Slower onset	Faster onset
**Therapeutic Efficacy**	Moderate efficacy	Superior efficacy
**Dosage Requirements**	Higher required dosages	Lower effective dosages
**Clinical Outcomes**	Moderate reduction in inflammation and pain	Greater reduction in inflammation, pain severity, and improved neurological outcomes
**Clinical Evidence**	Supported by preclinical studies and limited clinical trials [3,80]	Strongly supported by robust clinical and meta-analytic evidence [25,29,81]

PEA: palmitoylethanolamide; umPEA: ultramicronized palmitoylethanolamide; CNS: central nervous system; BBB: blood–brain barrier; BID: bis in die (twice daily).

**Table 2:** Clinical indications.

Clinical Condition	Recommended Dose (mg/Day)	Therapeutic Rationale	References
Neuropathic Pain and Fibromyalgia	600 mg BID	Enhances analgesia, reduces central sensitization	Paladini et al., 2016 [80]; Gabrielsson et al., 2016 [104]; Schweiger et al., 2019 [81]
Autoimmune and Inflammatory Diseases	600–1200 mg	Regulates immune overactivation, supports tolerance	Steels et al., 2019 [77]; Blake et al., 2006 [106]
Alzheimer’s and Parkinson’s Disease	600–1200 mg	Neuroprotection, slows progression, cognitive support	Scuderi et al., 2018 [25]; Beggiato et al., 2019 [5]
Stroke and Traumatic Brain Injury	600 mg BID	Reduces neuroinflammation, promotes recovery	Cordaro et al., 2016 [107]; Ahmad et al., 2012 [108]
Leaky Gut and Microbiome Support	300–600 mg	Restores gut barrier, modulates microbiota	Esposito et al., 2014; [73] Branković et al., 2024 [109]
Viral Infections and Immune Support	600–1200 mg	Enhances innate immunity, reduces inflammation	Re et al., 2021 [110]

**Table 3:** Clinical doses, therapeutic indications, and observed outcomes with umPEA in clinical practice.

Clinical Condition	Recommended Dosage	Therapeutic Context	Main Outcomes	Model Type	Mechanisms Investigated	References
Neuropathic Pain	600–1200 mg/day (divided into 2 daily doses)	Peripheral neuropathy, diabetic neuropathy, sciatic pain	Reduction in pain severity, improved quality of life, decreased analgesic usage	Clinical (human)	Anti-inflammatory, analgesic, mast cell stabilization	Paladini et al., 2016 [80]; Gabrielsson et al., 2016 [104]
Fibromyalgia	600–1200 mg/day	Chronic widespread pain, fatigue, muscle tenderness	Reduced pain intensity, improved sleep quality, reduced fatigue	Preclinical (mouse model of Alzheimer’s)	Anti-inflammatory, neuroprotective, glial modulation	Schweiger et al., 2019 [81]
Sciatica and Low Back Pain	600 mg twice daily	Radicular pain, chronic low back pain	Significant reduction in pain intensity, improved functional recovery	Preclinical (rat model of Parkinson’s)	Neuroprotection, antioxidant activity	Paladini et al., 2016 [80]; D’Amico et al., 2020 [111]
Pelvic Pain (Endometriosis)	600–1200 mg/day	Chronic pelvic pain, dysmenorrhea, endometriosis	Pain relief, reduced inflammation, improved daily activities	Preclinical (Alzheimer’s triple transgenic mouse)	Neuroinflammation modulation, astroglial regulation	Giugliano et al., 2013 [75]
Osteoarthritis	600 mg twice daily	Knee osteoarthritis, joint inflammation, and pain	Reduction in pain and joint stiffness, improved joint function	Clinical (human, endometriosis)	Pain modulation, anti-inflammatory action	Steels et al., 2019 [77]
Migraine and Primary Headache	1200 mg/day	Migraine, tension-type headache	Decreased attack frequency, reduced duration and intensity of headaches, reduced analgesic consumption	Clinical (human)	Analgesia, mast cell stabilization	Lang-Illievich et al., 2023 [105]; Gabrielsson et al., 2016 [104]
Neurodegenerative Disorders (Alzheimer’s, Parkinson’s)	600–1200 mg/day	Early cognitive decline, motor impairment	Neuroinflammation reduction, cognitive stabilization, improved motor function	Clinical (human)	Anti-inflammatory, pain relief	Scuderi et al., 2018 [25]; Beggiato et al., 2019 [5]
Postoperative Pain	600 mg twice daily	Post-surgical inflammation, pain management	Reduced post-operative pain severity, reduced reliance on opioid analgesics	Clinical (human)	Neuropathic pain relief	Paladini et al., 2016 [80]
